# Mobile cell-phones (M-phones) in telemicroscopy: increasing connectivity of isolated laboratories

**DOI:** 10.1186/1746-1596-4-19

**Published:** 2009-06-19

**Authors:** Livia Bellina, Eduardo Missoni

**Affiliations:** 1 Azienda Sanitaria Locale 6, Regione Sicilia, Via G. Cusmano 24, 90141 Palermo, Italy; 2 Centre for Research on Health and Social Care Management, CERGAS, Università Commerciale Luigi Bocconi, via Roentgen 1, 20136 Milano, Italy

## Abstract

**Background:**

The development of modern information telecommunication (ITC) technology and its use in telemedicine plays an increasingly important role in facilitating access to some diagnostic services even to people living in the most remote areas. However, physical and economical constraints in the access to broad band data-transmission network, still represent a considerable obstacle to the transmission of images for the purpose of tele-pathology.

**Methods:**

Indifferently using m-phones of different brands, and a variety of microscopic preparations, images were taken without the use of any adaptor simply approaching the lens of the mobile cell phone camera to the ocular of common optical microscopes, and subsequently sent via Multimedia Messaging Services (MMS) to distant reference centres for tele-diagnosis. Access to MMS service was reviewed with specific reference to the African information communication technology (ICT) market.

**Results:**

Images of any pathologic preparation could be captured and sent over the mobile phone with an MMS, without being limited by appropriate access to the internet for transmission (i.e. access to broad-band services). The quality of the image was not influenced by the brand or model of the mobile-phone used, but only by its digital resolution, with any resolution above 0.8 megapixel resulting in images sufficient for diagnosis.

Access to MMS services is increasingly reaching remote disadvantaged areas. Current penetration of the service in Africa was mapped appearing already available in almost every country, with penetration index varying from 1.5% to 92.2%.

**Conclusion:**

The use of otherwise already widely available technologies, without any need for adaptors or otherwise additional technology, could significantly increase opportunities and quality diagnostics while lowering costs and considerably increasing connectivity between most isolated laboratories and distant reference center.

## Background

The extension to all peoples of the benefits of medical knowledge is essential to the fulfilment of the enjoyment of the highest attainable standard of health as one of the fundamental rights of every human being.[1] The development of modern ITC technology and its use in tele-medicine plays an increasingly important role in facilitating access to some diagnostic services even to people living in the most remote areas, thanks to the possibility to analyse data transmitted from geographically very distant sources, [2] as in the case of tele-pathology.

However, complexity and costs of equipment, as well as physical and economical constraints in the access to broad band data-transmission network, still represent a considerable obstacle to the transmission of images for the purpose of tele-pathology. Even most recent extensive reviews of these limitations and the deriving challenges,[3] do not consider the possibility of capturing high quality images directly from the microscope, and transmitting them for distance diagnosis purposes using a mobile cell-phone (m-phone), without the need of any adaptor or additional technology. Based on the results of our testing, experience, and investigation on accessibility, we argue that the simple procedure we propose represents a far more appropriate and promising approach compared with those previously described.

## Methods

The images we collected originated from different brands and models of common optical microscopes, using ×10, ×25, ×40 and ×100 lenses according to the material to be observed, using usual microscopic techniques.

We tested the method on a variety of samples and preparations (wet or stained with routine methods) of biological material (blood, stools, urinary sediment, histologic, etc.).

Still images and short videos where captured with m-phones of a number of different brands, all with incorporated cameras and the possibility to send images via Multimedia Messaging Service (MMS). We tested the procedure using resolutions varying from 0.3 mega-pixels, up to 3.0 mega-pixels.

Images were taken without the use of any adaptor simply approaching the lens of the m-phone camera to the ocular of the microscope carefully centering the observed field, until the image would appear perfectly in focus on the screen of the mobile cell-phone, using hands to shield from external light interference.

The same technique was tested for comparison using a few models of digital cameras.

The captured image was sent directly using an MMS and read by the recipient both on his cell phone and downloading it on a Personal Computer (PC) for further digital amplification and analysis.

Both scientific and lay literature was extensively reviewed to compare results with other experiences and studies. Finally, in order to verify the usefulness of the proposed method in most remote and disadvantaged areas of the world, literature concerning the overall ICT market were also reviewed and data made publicly available by providers were analysed and mapped. Considering that Africa suffers the highest digital gap, we limited this research to that area of the world.

## Results

### Capturing and sending images

In April 2008, one of the authors, working as a pathologist on the Italian Island of Lampedusa, urgently needed to confirm a diagnosis of malaria from a blood sample of an African immigrant. With no other means at hand, she took a picture of the microscopic field using the camera incorporated in m-phone, without additional devices, and sent it via MMS for tele-diagnostic purposes to a reference center.

The excellent result obtained in such an unexpectedly easy way (with the distant reference center confirming the malaria case) stimulated the research and further testing of the method, both for diagnostic and didactic purposes.

**Figure 1:**
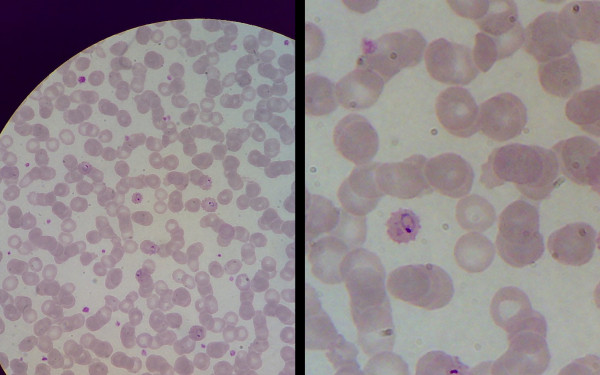
**Image captured with m-phone directly from microscope's ocular**. *Plasmodium falciparum *rings in erythrocytes, May-Grünwald-Giemsa stain. Without enlargement (left) and with m-phone camera incorporated zoom (x2.5) (right).

After some training, whatever the microscope or m-phone used, excellent images (both still images and videos) of any pathologic preparation could be captured and sent over the mobile phone with an MMS. Images could be easily downloaded on a computer both locally and at the recipient's place for better view and analysis. The quality of the image was not influenced by the brand or model of the m-phone used, but only by its digital resolution. In many cases even at 0.3 megapixel pictures consent diagnosis, however pictures taken with any resolution above 0.8 megapixel resulted in images appropriate for diagnostic purposes. M-phones incorporated camera's zoom facility greatly enhanced the capacity even at the lowest resolutions. Figure 1 shows the same microscopic field taken at 0.8 megapixel with or without zoom (2.5×). Intra-erythrocytic *Plasmodium falciparum *rings can easily be detected and described.

The use of videos allowed additional capabilities, as in the case of the diagnosis of trophozoites of sporozoan intestinal parasites in wet stool preparations.

In additon of lacking the possibility to directly transfer images via MMS, thus requiring to that purpose a computer with access to a broad-band internet connection, capturing images with any of the tested digital cameras (without the use of any adaptor) resulted to be much more cumbersome, due to the dimension of the lenses and the difficulty to ensure correct focussing, as well as preventing interference from external light.

### Accessibility to MMS services and trends

In the last ten years mobile telephony increased three times faster than other telecommunications systems, such as landline phones and the internet.[4] Worldwide, between 2000 and 2008 annual m-phone subscriptions experienced an average annual increase of 24%, and current historical economic recession does not seem having significantly altered the trend.[5]

According to International Telecommunication Union (ITU) in 2008 mobile phone users were 61.1% of world population, compared with 18.9% of landline phone users, 23% of internet users and respectively 5% and 6.1% of mobile broad band and fixed broad band subscribers. A closer look to developing countries shows that by the end of 2007 mobile cellular subscriptions had reached close to 40% of the population, representing 64% of world subscriptions, whereas in 2002 mobile phone users in developing countries where only 44%.[4]

**Figure 2:**
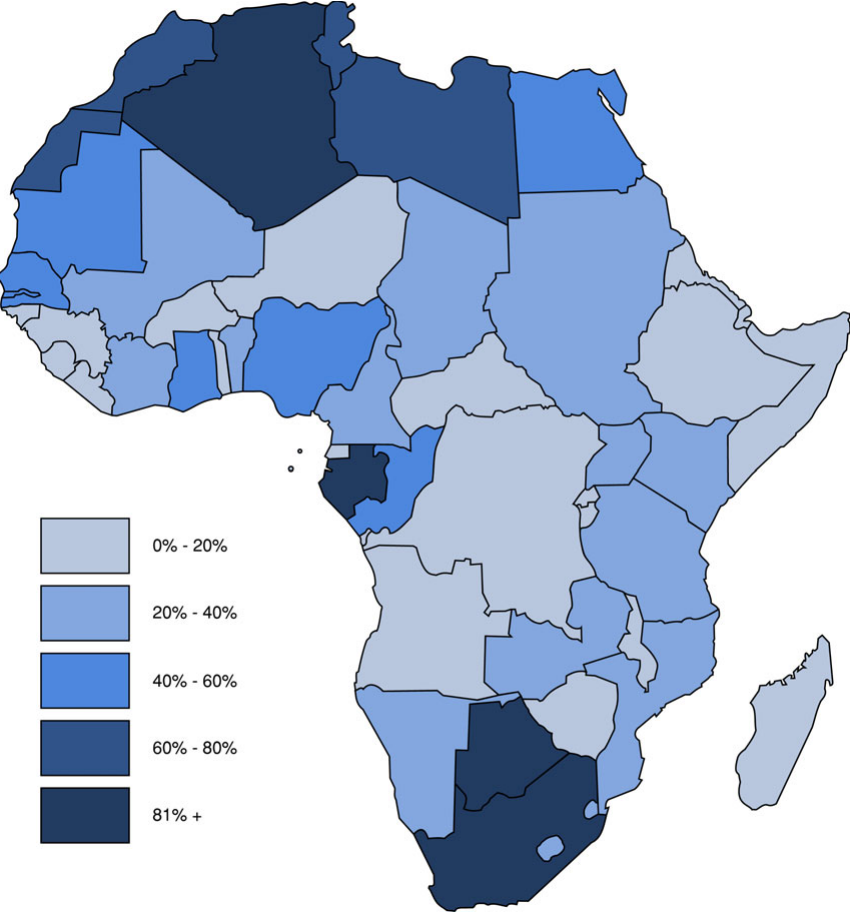
**M-phone and MMS penetration in Africa**. M-phone subscriptions with declared availability of MMS, per 100 inhabitants. Map elaborated by the authors based on last available information (2007–2009).

Mobile phone penetration does not necessarily mirror the access to MMS service. However the penetration of the latter seems to be following a similar trend, increasingly reaching remote and disadvantaged areas of the world. Combining available data from multiple sources regarding m-phone subscriptions and MMS declared availability,[6] we could map the current penetration of the service, in Africa, where MMS appears to be already available in almost every country in Africa. Latest available information (2007–2008) shows penetration index (subscriptions per 100 inhabitants) varying from 1.5% (Ethiopia, 2007) to 92.2% (South Africa, 2008) (Figure 2).

## Discussion

Surprisingly, notwithstanding the easiness of the method, the use of m-phones for both capture and transmission of microphotographic images of medical relevance was never reported in scientific literature. It must be noted that, even the use of a m-phone incorporated camera to simply capture pictures from optical devices is scarcely described. Through search on the internet we could find only one reference to that approach in lay media [7] and only one article referring to it for medical application in scientific literature,[8] none explicitly referring to the use of MMS for distance transmission. The advantage of instant capture and transmission of medical images via MMS has been recently suggested also in a urological setting, [9] and the development of a searchable archive of camera phone images for medical education, suggests additional potential of the use of m-phones and MMS for medical imaging. [10]

On the other hand there is wide reference and promotion on the internet of optical adaptors and more complex techniques for the capture and transmission of microphotography images which seems to elicit the interest of the market,[11] whereas a microscope is normally already available in any medical laboratory and as we show an adaptor is not needed.

Besides the arguable technological appropriateness and cost of additional equipment (cameras and/or adaptors, computer, etc.), physical and economical constraints in the access to broad band data-transmission network, represent a major obstacle to the transmission of images for the purpose of tele-pathology in most remote and disadvantaged areas of the world. These limitations and the deriving challenges were recently extensively reviewed by Alfaro and Roca, who defined the minimum requirements for a system of image transmission,[3] however without mentioning MMS as an alternative to the internet for transmission.

The potential of "mHealth" for health care delivery in India was described in a meeting held in Bellagio in 2008.[12] In fact, the possibility to both capture and send images taken from the microscope, and by extension from any optical eyepiece of other medical devices (enteroscope, colposcope, etc.), with an MMS via the mobile cell-phone network represent an enormous breakthrough in terms of simplicity and access, given the penetration of the mobile network and transmission costs, in comparison to the landline broadband network or satellite connection on which the internet needs to rely.

## Conclusion

Universal access to health requires political, rather than technological solutions. However, a wide dissemination of the use of otherwise already available technologies (both m-phones and optical microscopes are largely disseminated in health centers all over Africa), without any need for adaptors or otherwise additional technology, could significantly increase opportunities and quality diagnostics while lowering costs and considerably increasing connectivity between most isolated laboratories and distant reference center.

## Competing interests

The described method has been filed for patent by LB in April 2008, with the sole purpose to protect the idea from commercialization and consent its free use and dissemination.

## Authors' contributions

LB conceived the proposed technique and experimented it in the field, EM verified the quality of received images and their appropriateness for diagnostic purposes. Both authors contributed equally in the bibliographic research and organization of data, as well as in the drafting of the manuscript.
